# Changes in standard of candidates taking the MRCP(UK) Part 1 examination, 1985 to 2002: Analysis of marker questions

**DOI:** 10.1186/1741-7015-3-13

**Published:** 2005-07-18

**Authors:** IC McManus, J Mollon, OL Duke, JA Vale

**Affiliations:** 1 Dept of Psychology, University College London, Gower Street, London WC1E 6BT, UK; 2 MRCP(UK) Central Office, 11 St Andrews Place, Regents Park, London NW1 4LE, UK

## Abstract

**Background:**

The maintenance of standards is a problem for postgraduate medical examinations, particularly if they use norm-referencing as the sole method of standard setting. In each of its diets, the MRCP(UK) Part 1 Examination includes a number of marker questions, which are unchanged from their use in a previous diet. This paper describes two complementary studies of marker questions for 52 diets of the MRCP(UK) Part 1 Examination over the years 1985 to 2001 to assess whether standards have changed.

**Methods:**

Study 1, which used routinely collected information on the performance of 4405 marker items, used a statistical method to assess changes in performance across diets. Study 2 compared performances of individual candidates on 28 individual marker items that were shared by the 1996/2 and 2001/3 diets.

**Results:**

Study 1 found evidence that candidate performance on the MRCP(UK) Part 1 Examination showed a gradual improvement over the period 1985 to 1997, which was followed by a sharp decline in performance until 2001. The 'dog-leg' in performance at 1997/3 was not an artefact of changed Examination Regulations, mix of UK and overseas candidates, or time from qualification until taking the Examination. Study 2 confirmed that performance in 2001/3 was significantly worse than in 1996/3, that the poorer performance was found in graduates of UK medical schools, and that candidates passing the Examination in 2001/3 performed less well than those passing in 1996/2.

**Conclusion:**

There has been a decline in the performance of graduates from UK medical schools taking the MRCP(UK) Part 1 examination. The reasons for this are not clear, but the finding has implications for medical education, and further studies are needed of performance in other postgraduate and undergraduate examinations. The use of norm-referencing as the sole method for setting the pass mark over this period meant that candidates passing the MRCP(UK) examination also had a lower standard. The MRCP(UK) Part 1 and Part 2 examinations now have their standard set by criterion-referencing.

## Background

The role of postgraduate medical examinations is to set standards of practice and thus to assure the public and the medical profession that doctors have the knowledge and expertise required to diagnose and treat patients, and to progress in their medical careers. An important part of running "high-stakes" qualifying examinations is the setting of a pass mark, and many postgraduate examinations in the UK have relied on *norm-referencing*, in which a fixed proportion of candidates passes at each occasion [1].

Although administratively straightforward, norm-referencing has several problems. The absolute performance across different diets of the exam varies, firstly due to asking questions of different difficulty, and secondly as a result of candidates differing in their ability. These factors of question difficulty and candidate ability are entirely confounded when only overall examination performance is considered, and norm-referencing cannot assess whether candidate ability varies from occasion to occasion. The result, as was shown in an important and influential analysis of the American Board of Internal Medicine's examination, was a slide in standards over the time-period 1983 to 1988 [2]. On that basis the Board implemented a process of *criterion-referencing*, in which examiners set a pass mark by assessing the content of each individual question on the examination. Having said that, we also acknowledge that there are arguments in some situations for the use of norm-referencing [3], and as a result there are even stronger arguments for the use of compromise methods [4,5].

In this paper we primarily wish to assess whether the absolute standard of candidates taking the MRCP(UK) examination changed over the period 1985 to 2002, when the format of the examination and the method for setting its pass mark were relatively constant. The assessment of the true ability of candidates, independently of question difficulty, requires a process of *equating *to establish whether candidates on one occasion are of equivalent ability to those on another occasion. Statistical equating can be carried out if *marker questions *are available, the same questions being used on two different diets [6].

### Background to Study 1

In this paper we describe two complementary analyses of marker questions used in the MRCP(UK) Part1 Examination between 1985 and the first diet of 2002 (2002/1). Three diets of the Examination were held each year, and in all diets the pass rate was norm-referenced at 35% of those candidates taking the Examination on their first four attempts at UK examination centres.

Two separate studies are described:

**Study 1**: *Analysis of aggregate performance of marker questions, 1988/1 to 2002/1*.

**Study 2**: *Comparison of 1996/2 and 2001/3 diets*

Although Study 2 follows on from Study 1, conceptually and in practice, the requirements of the journal are that the method of each is presented before the results of each, and then the discussion of each. This is somewhat confusing, and in particular it is necessary to present the background and justification to study 2 before the results of Study 1. Readers who are confused by this layout are referred to the **Additional File**, where the text of the paper is ordered in a more logical format.

### Background to Study 2

As will become apparent, Study 1 demonstrates that performance on marker questions declined during the period 1997 to 2002. However there are several possible interpretations of that result, not least because the data are aggregated across all candidates and do not allow analysis of sub-groups of candidates, such as UK graduates on first and subsequent attempts. Study 2 therefore analysed raw data at the level of responses to individual questions from individual candidates, thereby allowing a detailed comparison of the two diets.

## Methods

### Methods: Study 1

The format of the Examination, which was held three times a year, was unchanged until 2002/1, consisting of 60 five-part Multiple True-False questions (a total of 300 items). Negative marking was used in scoring the examination, and results were expressed as a 'corrected percentage correct', which takes guessing into account. The only substantive change in the Examination Regulations was that candidates were allowed to make an unlimited number of attempts from 1999/2 onwards, whereas previously they had been limited to four attempts.

Marker items were defined as any items included for a second time in any of the 43 diets of the MRCP(UK) Part 1 Examination held between 1988/1 and 2002/1, and which had been used in the previous diet with the stem, the item and the correct answer unchanged. Documents for tracing markers prior to 1988 were not readily available. The dates of the two diets, and the proportion of candidates getting each item right, wrong or not answering it, were recorded for each marker item.

### Statistical analysis: Study 1

The central issue in a marker question analysis is whether, on average, aggregate performance on items has increased or decreased between the first and second usage. By assessing how such a change relates to the dates when the items were used, one can estimate the changing overall performance of candidates in the Examination. The study is a variant of an incomplete paired comparison design [7,8].

If the percentages of candidates who get an item correct or wrong on the first and second occasion are *c*_*1*_, *w*_*1 *_and *c*_*2*_, *w*_*2*_, then the change in performance of the item is:

*Δ *= (*c*_*2 *_- *w*_*2*_) - (*c*_*1 *_- *w*_*1*_)

It should be noted that *Δ *is independent of the proportion of candidates choosing not to answer an item since those not answering are effectively neutral, neither gaining a mark from a correct answer nor losing one due to a wrong answer. Given the different performance of the marker questions at different diets, one can reconstruct the changing true ability of the candidates by a process analogous to triangulation, a process that can be carried out using multiple regression (for a technical explanation see below). The method takes one arbitrarily chosen date as the reference category (the first diet of 2000 was used) and estimates the ability of the candidates at each other diet relative to the reference diet. The estimates, like all regression coefficients, have a standard error and confidence intervals.

### Statistical method, Study 1: details of technique

Although there are many articles and books devoted to test equating, they usually consider only the problem of a large set of overlapping items occurring in two adjacent diets of an examination. The marker questions for Part 1 are large in number but are distributed across many diets, making a different problem for estimation. It is interesting to note that the regression solution described here is very general in its applicability, and was previously used in an entirely different context [9].

The exam is taken on *n *occasions. Let the standard of the candidates vary, such that for diet *d*, the true standard, relative to some arbitrary reference diet, *r*, is *s*_*d *_(i.e. *s*_*r *_= 0). If the same item (question) were to be used on every diet, then on diet 1, a proportion *s*_*1 *_would get the item correct, on diet 2 *s*_*2 *_would get the item correct, etc.

A diet has a variable number of marker items, which have been used unchanged in a number of previous diets. An individual marker item, *m*, in diet *k*, will have been used previously in, say, diet *j*. The statistical analysis is required to derive the true standard of the candidates at the various diets, *s*_*i *_(*i *= 1, *n*; *s*_*r *_= 0), from the performance on the marker items.

Let the difference in performance on marker item *m *between diets *j *and *k *be expressed as *Δ*_m_. In the specific case of a true-false examination with negative marking, let *c*_*j *_% get the item correct at time *j*, *w*_*j *_% get the item wrong at time *j*, and *na*_*j *_% not answer the item at time *j *(and with equivalent symbols for time *k*). *Δ*_m _is then calculated as:

*Δ*_m _= (*c*_*k*_-*w*_*k*_)-(*c*_*j*_-*w*_*j*_)

Note that if the performance of the candidates on the question is the same at diets *j *and *k *then

*Δ*_m _= 0.

A series of dummy variables, *v_1 _*to *v*_*n*_, is then created for each marker variable, which take the values :

*v*_*j *_= -1

*v*_*k *_= +1

*v*_*p *_= 0 [*p *= 1, *n*; *p* not equal to *j*, *p* not equal to *k *]

*s*_*d *_(*d *= 1,n) can be estimated using multiple regression, where *Δ*_m _is the dependent variable, and the predictor variables are *v_1 _*to *v*_*n *_(excluding the reference diet, *v*_*r*_). The unstandardised regression coefficients of *v*_*d *_(*d *= 1,n) are then the estimates of *s*_*d *_(*d *= 1,n), with *s*_*r *_= 0, and the standard errors of the regression coefficients are the standard errors of the estimates of *s*_*d *_(*d *= 1,n).

SPSS version 11.5 was used for statistical analysis of the data. For statistical analysis, diets 1, 2 and 3 in a year were set at 0, .33 and 0.67 of the year.

### Methods: Study 2

**Figure 1:**
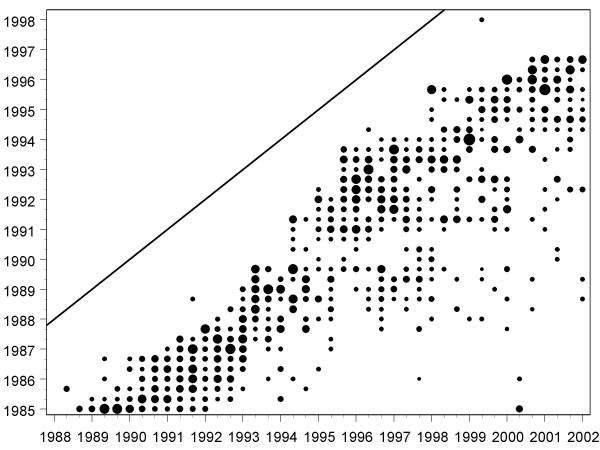
Date of previous setting (vertical axis) of marker questions used in various diets (horizontal axis). The horizontal axis shows a particular diet of the exam, and the vertical axis the previous diets from which marker items were taken. Size of points is proportional to the number of questions.

**Figure 2:**
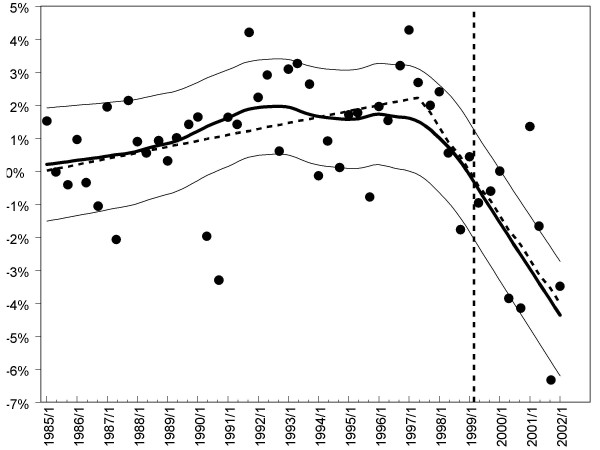
The estimated true ability of candidates taking the examination at each diet. The solid points are the estimates from the regression (*s*_*d*_). The thick black line is a fitted lowess line (locally weighted regression), and the thin black lines show lowess lines through the one standard error confidence intervals for the points. The thick dashed line is the 'dog-leg' curve (see text). The vertical dashed line indicates the date when unlimited attempts were allowed in the Part 1 examination.

Raw data were available only for the 1996/2 diet and the diets from 1997/1 onwards, and of these only the 1996/2 and 2001/3 diets shared sufficient marker items for analysis (see figure 1). Since the 1996/2 diet took place just before the apparent decline in performance seen in figure 2, whereas 2001/3 took place afterwards, a comparison is appropriate.

The 1996/2 and 2001/3 diets had 28 items in common, which were scored on the basis of +1 for a correct answer, -1 for a wrong answer and 0 for no answer, giving a '*marker score*' expressed as a corrected percentage score.

For the 1996/2 diet the pass mark, defined by the MRCP(UK) Part 1 Examining Board as the performance of the top 35% of candidates taking the Examination in the UK on their 1^st ^to 4^th ^attempts, was 54.84%, and for the 2001/3 diet it was 48.50%. For comparative purposes only, candidates are here divided into four groups, 'Fail, 'Bare Fail', 'Bare Pass' and 'Pass'; the threshold between Bare Fail and Bare Pass was the pass mark itself, and the thresholds between Fail and Bare Fail and between Bare Pass and Pass were set five absolute percentage points below or above the pass mark. The thresholds defining the four groups are therefore 49.84%, 54.84% and 59.84% for the 1996/2 diet, and 43.50%, 48.50% and 53.50% for the 2001/3 diet.

All analyses were restricted to 'UK graduates', defined as those with primary registrable qualifications from United Kingdom medical schools.

## Results

### Results: Study 1

Altogether 5332 marker questions were used in the 43 diets held between 1988/1 and 2002/1, and these had previously been used on diets extending back to 1972. Although in principle the statistical method can assess standards outside of the range of the diets actually assessed, a preliminary analysis suggested the process was unstable prior to about 1985, with large standard errors of the estimates. Analysis is therefore restricted to the 4405 marker questions that provided information on the performance of the 52 diets held between 1985/1 and 2002/1.

Figure 1 shows the timing of the two diets in which a marker item had been used. On average each diet, which consisted of 300 items, contained 124 marker items (41.3%), (SD 23.4; range 72–173). Marker items had on average been used 5.7 years previously (i.e. 17 diets; SD 2.22, range 1.33–24.33). The average number of marker items in any one diet that came from the same previous diet was 10.4 (SD 7.6, range 1 to 48).

The individual points in figure 2 show the estimated ability of candidates at each diet from 1985/1 to 2002/1, *s*_*d*_, calculated by regressing the dummy variables *v*_*1 *_to *v*_*n *_on the values of *Δ*_m_. The ability of candidates increased slowly but consistently between 1985 and about 1996, after which ability appears to decline fairly steeply. That hypothesis was formalised by using non-linear regression to fit two straight lines to the data, each with its own independent slope, with a 'dog-leg' mid-way through the data, the date of the dog-leg itself being a free parameter. The dog-leg curve shown in figure 2 fits the data well (R^2 ^= .468), and is a significant improvement over a simple linear regression (F(2,48) = 18.28, p < 0.001). The inflexion of the dog-leg is at 1997.3 (equivalent to the 1997/2 diet), with 95% confidence intervals of 1996.0 to 1998.6 (equivalent to diets 1996/1 to 1998/3). The slope before 1997 is +0.18%/year (95% CI 0.03% to 0.33%), and the slope after 1997 is -1.34%/year (95% CI -1.93% to -0.75%).

### Results: Study 2

The 1996/2 diet was taken by 2132 candidates of whom 852 were UK graduates. The 2001/3 diet was taken by 2051 candidates of whom 557 were UK graduates. The 28 marker items were combined into a single scale for which alpha was 0.593, which is equivalent, using the Spearman-Brown formula [10], to a reliability of 0.940 for a full-length 300 item test, somewhat higher than the mean reliability across 54 diets of the examination as a whole of 0.865 [11].

### Overall performance on marker items: Study 2

**Figure 3:**
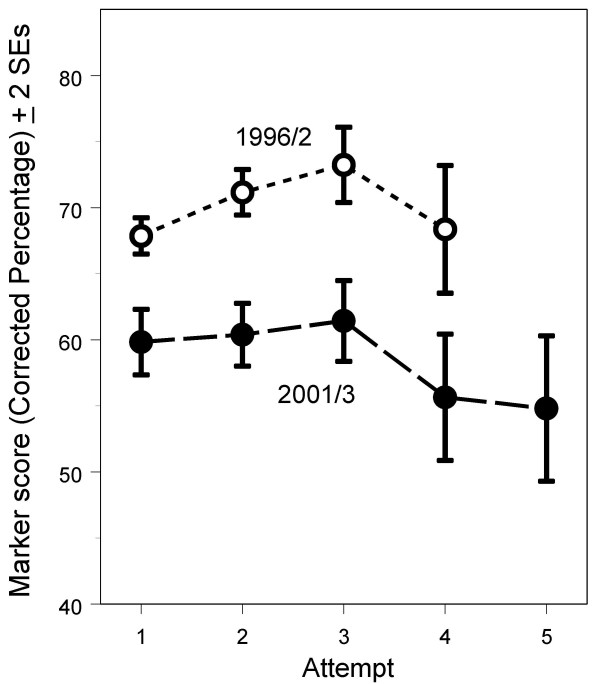
Overall performance on the 28 marker items for UK graduates taking the 1996/2 and 2001/3 diets of the examination, on their first and subsequent attempts. Until diet 1999/2 candidates were only allowed to take the exam four times, whereas after 1999/2 they were allowed unlimited attempts. Fewer than 20 candidates were on their 6^th ^or higher attempt, and they have been omitted from the analysis.

In the 1996/2 diet, the 852 UK graduates have a mean marker score of 69.4 (SD 14.5), compared with a mean marker score of 59.6 (SD 15.8) for the 557 UK graduates in the 2001/3 diet (t = 11.92, 1407 df, p < 0.001). The average mark in 2001/3 was therefore 14.1% lower than in 1996/2 when precisely the same items are compared. Detailed analyses of items answered correctly, incorrectly or not answered showed that the 2001/3 candidates had answered fewer of the 28 items correctly (20.54 vs 22.40; p < 0.001) and more of the items incorrectly (3.84 vs 2.98; p < 0.001), and more were not answered (3.84 vs 2.61; p < 0.001). The reduced performance in the 2001/3 diet was independent of whether candidates were taking the Examination on their first, second or subsequent attempts (see Figure 3; ANOVA: diet F(1,1362) = 96.7, p < 0.001; attempt F(4,1362) = 3.63, P = 0.006; interaction F(3,1362) = 1.22, P = 0.300).

### Performance in relation to pass mark: Study 2

**Figure 4:**
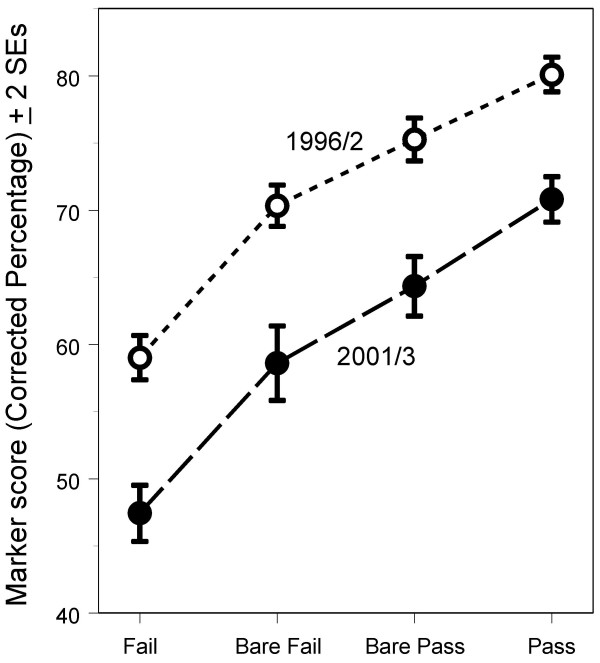
Performance of UK candidates on the marker items in relation to overall examination performance (see text for details of Fail, Bare Fail, Bare Pass, and Pass). Points are plotted ± 2 standard errors.

Analysis of the marker scores of candidates in each diet according to candidates' overall performances in the Examination showed lower scores in 2001/3 than in 1996/2 for all ability levels, including those who had passed the exam (figure 4: ANOVA: diet F(1,1401) = 255.3, p < 0.001; ability group F(3,1401) = 237.30, P < 0.001; interaction F(3,1401) = 0.705, P = 0.549).

### Performance on individual marker items in 1996/2 and 2001/3: Study 2

**Figure 5:**
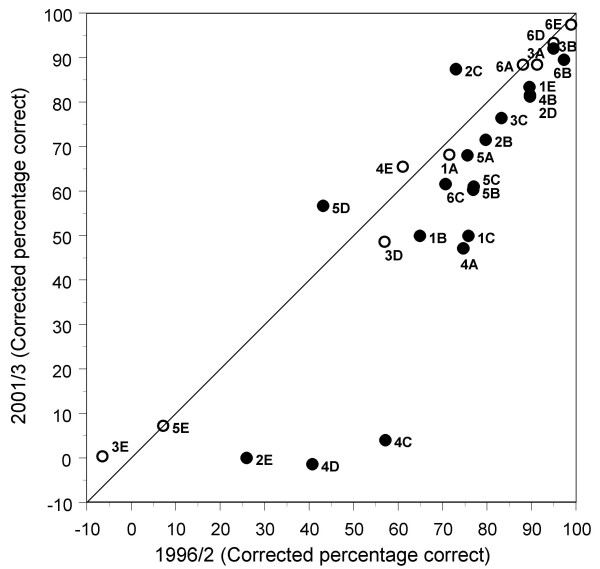
Performance of the 28 individual marker items in the 1996/2 and 2001/3 diets. The diagonal line is the point of equality; points below the line represent items for which performance is worse in 2001/3 than in 1996/2. Solid points are statistically significant (p < .05), and open points are non-significant. Code numbers of items are those shown in table 1.

In 2001/3, UK candidates performed significantly less well (p < 0.05) on 17 of the 28 individual marker items (table 1) and better on 2 items, and performance was not significantly different for 9 items (see Figure 5). The median change in performance was -7.65% (inter-quartile range -1.42% to -15.73%).

**Table 1:** Performance of UK graduates on marker items in the 1996/2 and 2001/3 diets (N = 852 and 555 respectively). Significance tests are shown for each item.

1: Recognised features of infectious mononucleosis include:		1996/2 Q.13	2001/3 Q.15
A: Petechial haemorrhages on the palate [True] *(Kendall's tau = -0.038, p = 0.153)*	Right	81.0%	77.7%
	NA	9.5%	11.9%
	Wrong	9.5%	10.5%
B: a vesicular rash on the neck and trunk [False] *(Kendall's tau = -0.097, p < 0.001)*	Right	76.1%	67.4%
	NA	12.8%	15.1%
	Wrong	11.2%	17.5%
C: aseptic meningitis [True] *(Kendall's tau = -0.178, p < 0.001)*	Right	82.5%	67.4%
	NA	10.8%	15.1%
	Wrong	6.7%	17.5%
E: raised serum aspartate aminotransferase activity [True] *(Kendall's tau = -0.080, p = 0.003)*	Right	91.4%	86.3%
	NA	6.7%	10.8%
	Wrong	1.9%	2.9%
Item D was not a marker item			

2: A patient with chronic osteitis of the femur associated with a discharging sinus has a haemoglobin of 10.5 g/dL, MCV 78 fl, MCH 30 pg, WBC 10.8 × 10^9^/L, platelets 420 × 10^9^/L, and a normal blood film. The following statements are correct:		1996/2 Q.17	2001/3 Q.21

B: Antibiotic therapy is the probable cause of the blood picture [False] *(Kendall's tau = -0.078, p = 0.004)*	Right	85.8%	79.6%
	NA	8.1%	12.3%
	Wrong	6.1%	8.1%
C: C-reactive protein levels will be normal [False] *(Kendall's tau = +0.118, p < 0.001)*	Right	83.3%	91.5%
	NA	6.3%	4.3%
	Wrong	10.3%	4.1%
D: Parenteral iron therapy would be valuable in treating the anaemia [False] *(Kendall's tau = -0.080, p = 0.004)*	Right	92.6%	87.9%
	NA	4.5%	5.4%
	Wrong	2.9%	6.7%
E: Abundant stainable iron will be found in bone marrow macrophages [True] *(Kendall's tau = -0.160, p < 0.001)*	Right	45.5%	27.9%
	NA	34.9%	44.1%
	Wrong	19.6%	27.9%
*NB *Item D was not a marker item			

3: Recognised features of acute poisoning due to theophylline include:		1996/2 Q.27	2001/3 Q.24

A: vomiting [True] *(Kendall's tau = -0.040, p = 0.139)*	Right	92.7%	90.5%
	NA	5.8%	7.6%
	Wrong	1.5%	2.0%
B: convulsions [True] *(Kendall's tau = -0.056, p = 0.043)*	Right	95.9%	93.3%
	NA	3.2%	5.4%
	Wrong	0.9%	1.3%
C: supraventricular tachycardia [True] *(Kendall's tau = -0.086, p = 0.001)*	Right	88.4%	81.8%
	NA	6.5%	12.8%
	Wrong	5.2%	5.4%
D: hypokalaemia [True] *(Kendall's tau = -0.044, p = 0.093)*	Right	69.0%	65.8%
	NA	18.9%	17.1%
	Wrong	12.1%	17.1%
E: metabolic acidosis [True] *(Kendall's tau = +0.040, p = 0.110)*	Right	26.9%	30.8%
	NA	39.8%	38.7%
	Wrong	33.3%	30.5%

4: Recognised features of Wolff-Parkinson-White syndrome include:		1996/2 Q.33	2001/3 Q.33

A: an accessory connection between atria and the atrioventricular node [False] *(Kendall's tau = -0.177, p < 0.001)*	Right	85.7%	71.1%
	NA	3.3%	4.9%
	Wrong	11.0%	24.0%
B: prolonged PR interval [False] *(Kendall's tau = -0.096, p = 0.001)*	Right	94.4%	89.0%
	NA	0.9%	3.6%
	Wrong	4.7%	7.4%
C: prolonged QRS complex [True] *(Kendall's tau = -0.286, p < 0.001)*	Right	76.2%	47.5%
	NA	4.8%	9.0%
	Wrong	19.0%	43.5%
D: paroxysmal ventricular tachycardia [False] *(Kendall's tau = -0.223, p < 0.001)*	Right	65.8%	41.7%
	NA	9.0%	15.2%
	Wrong	25.1%	43.1%
E: dominant R waves in lead V1 of the ECG [True] *(Kendall's tau = +0.032, p = 0.213)*	Right	71.1%	74.0%
	NA	18.8%	17.5%
	Wrong	10.1%	8.5%

5: The following clinical features suggest an organic basis for psychiatric symptoms:		1996/2 Q.40	2001/3 Q.41

A: disorientation in time [True] *(Kendall's tau = -0.074, p = 0.006)*	Right	86.2%	80.0%
	NA	3.3%	8.1%
	Wrong	10.6%	11.9%
B: visual hallucinations [True] *(Kendall's tau = -0.122, p < 0.001)*	Right	86.9%	77.3%
	NA	3.2%	5.8%
	Wrong	10.0%	17.0%
C: mutism [False] *(Kendall's tau = -0.150, p < 0.001)*	Right	84.0%	70.6%
	NA	8.9%	19.9%
	Wrong	7.0%	9.6%
D: inability to retain new information [True] *(Kendall's tau = +0.071, p = 0.005)*	Right	67.0%	72.9%
	NA	9.2%	10.8%
	Wrong	23.8%	16.2%
E: perseveration [True] *(Kendall's tau = -0.004, p = 0.885)*	Right	45.3%	39.0%
	NA	16.5%	29.2%
	Wrong	38.1%	31.8%

6: Characteristic features of schizophrenia include:		1996/2 Q.43	2001/3 Q.40

A: memory impairment [False] *(Kendall's tau = +0.009, p = 0.725)*	Right	91.3%	91.9%
	NA	5.4%	4.7%
	Wrong	3.3%	3.4%
B: auditory hallucinations in clear consciousness [True] *(Kendall's tau = -0.125, p < 0.001)*	Right	98.5%	93.9%
	NA	0.4%	1.8%
	Wrong	1.2%	4.3%
C: incongruity of affect [True] *(Kendall's tau = -0.085, p = 0.001)*	Right	81.9%	73.8%
	NA	6.8%	13.9%
	Wrong	11.3%	12.3%
D: feelings of panic in buses and shops [False] *(Kendall's tau = -0.037, p = 0.178)*	Right	96.5%	94.9%
	NA	2.0%	3.4%
	Wrong	1.5%	1.6%
E: a feeling of being under the influence of an external force [True] *(Kendall's tau = -.038, p = 0.185)*	Right	99.1%	98.2%
	NA	0.7%	1.1%
	Wrong	0.2%	0.7%

## Conclusion

### Conclusions: Study 1

In the analysis of marker items in Study 1, candidates showed a gradual improvement in performance on the MRCP(UK) Part 1 examination for the twelve years between 1985 and 1997. Such a change could either result from a genuine increase in candidates' knowledge or from candidates becoming more 'test-wise', perhaps due to attending crammer courses and becoming more aware of questions from previous papers. However, the latter explanation seems unlikely in view of the sharp decline in performance of candidates after 1997.

If the decline in performance after 1997 reflects decreasing candidate ability, then that has important implications for medical education and training, and possible confounders and artefacts must be excluded. Separate analyses, not reported in full here but available in the **Additional File**, have investigated the following possibilities:

#### 1. Change in number of attempts allowed

From 1999/2 onwards, candidates were not restricted to four attempts at the Examination. Figure 2, however, shows that the decline in performance started well before that date.

#### 2. Changes in strategy in relation to negative marking

The MRCP(UK) Part 1 examination was negatively marked, wrong answers incurring a greater penalty than unanswered questions. If candidates changed their strategy, that may have produced an apparent decline in performance. We have modelled the number of not-answered questions, and although this did show a decline in between 1990 and 1993, the timing was unrelated to the change shown in Figure 2.

#### 3. Changes in the mix of candidates taking the Examination

Because the marker question statistics recorded in the Examination records are based on aggregate statistics for all candidates taking the examination, changes in the mix of candidates could cause a change in marker question performance. We have looked at the relative proportion of UK and overseas graduates, the numbers of candidates on first, second, third, fourth and later attempts, and the time between qualification and taking the MRCP(UK) Part 1 Examination, and although secular trends are visible, none shows a sudden change in 1997. (See **Additional File**).

This study of 4,405 marker items suggests the standard of candidates taking the Part 1 MRCP(UK) Examination may have changed over time, rising gradually until about 1997, and then declining rather more rapidly. The discontinuity around 1997 does not seem to be related to any obvious change in the structure of the Examination or the composition of the candidates taking it.

### Conclusion: Study 2

Study 2 examined the performances of individual candidates on marker items and confirms the earlier finding that performance dropped between 1996/2 and 2001/3. In particular, fewer UK graduates who passed the Examination in 2001/3 gave correct answers than had equivalent candidates in 1996/2. The median change in performance on items of -7.75% was similar to the expected change of -7.1%, based on the -1.34% per year shown in figure 2. The results of figure 3 are probably not therefore distorted or biased by inadvertent differences in candidate mix between the diets.

We found that the performances on individual marker items between 1996/2 and 2001/3 had dropped for 21 of the 28 items. None of the questions is about recondite, obscure or unimportant areas of knowledge for a general physician in training, and none of the changes are likely to reflect changes in the importance of knowledge, in understanding of disease mechanisms, or in treatment strategies. They are therefore acceptable marker questions. The largest decreases were on the electrocardiography and anatomy of the Wolff-Parkinson-White syndrome, aseptic meningitis in infectious mononucleosis, and bone marrow biopsy findings in the anaemia of chronic infection, all of which are important clinical problems. The only two significant increases in knowledge are on questions concerning C-reactive protein (a relatively recently introduced clinical test), and on the diagnosis of organic brain disease.

### Discussion: General

These two complementary studies have implications specifically for postgraduate medical examinations, and more generally for undergraduate medical education. The studies provide evidence that there was a sudden, relatively steep decline in the performance of candidates passing the MRCP(UK) Part 1 Examination between 1997 and 2002, which was not an artefact of changes in the mix of overseas and UK candidates, or changes in the time after qualifying of first or subsequent sittings of the examination. Study 2 confirmed that the decline had taken place in doctors graduating from UK medical schools.

#### i). Implications for standard setting in postgraduate medical examinations

The MRCP(UK) examination sets a standard for professional clinical practice in the UK. Our use of marker questions for assessing the standard across diets parallels a study in 1989, which described the falling standard of candidates passing the American Board of Internal Medicine (ABIM) examination [2] (although there are differences in the way marker questions were used). In both the ABIM examination and the MRCP(UK) Part 1 Examination the declining standard of candidates passing the examination probably arose from reliance on the sole use of norm-referencing for standard setting. Any other examination relying solely on norm-referencing may also be vulnerable to the same problem.

The MRCP(UK) Part1 and Part 2 written Examinations have recently changed their format, the Part 1 Examination now consisting entirely of 'best-of-five' questions. The MRCP(UK) Part 1 and Part 2 Examinations both carry out standard-setting by a process incorporating criterion referencing using the Angoff technique [12,13], with the pass mark itself set by the Hofstee compromise technique [4], which reduces the likelihood of large short-term swings in the pass rate. The pass rates in the three MRCP(UK) Part 1 Examination diets of 2003 for candidates on their first four attempts sitting the exam at UK centres were 31.5%%, 33.4% and 32.3%, somewhat less than the 35% that would have occurred using norm-referencing. Performance in the MRCP(UK) Part 1 Examination is continuing to be monitored by the use of marker questions.

#### ii). Implications for undergraduate and postgraduate medical education

The decline in performance of candidates from UK medical schools taking the MRCP(UK) Part 1 Examination raises questions extending beyond the Part 1 Examination itself. The examination can be taken eighteen months after graduation, and a high proportion of UK graduates take it at the earliest possible time, when they typically have five or six years of undergraduate education, a year of PRHO posts, and six months of SHO training. Several explanations need to be considered for the changes in standard that we have found.

##### i. Changing relevance of the examination questions

Topics once perceived as central to medical training may now no longer be important to modern medical practice. If the marker questions used were out-of-date then that may explain the apparent decline. However, not only is the content of marker questions always approved by MRCP(UK) Part 1 Examining Board before each inclusion in the Examination, but the questions shown in table 1 clearly relate to core conditions and their underlying disease mechanisms, and hence changes cannot be shrugged off as resulting from irrelevant or outmoded questions.

##### ii. Changing career patterns of graduates

The present results relate only to one examination, the MRCP(UK) Part 1 Examination, albeit an exam taken by over 30% of UK graduates. Corroboration of the present findings from other UK postgraduate examinations is desirable, in order to assess the generality of the findings. It is possible that around 1997 more able UK graduates candidates decided they no longer wished to take the MRCP(UK) Part 1 Examination, and instead took other career paths (and that seemed to be the explanation for the declining standard in the ABIM examination [2]). Although perhaps unlikely, the possibility can be assessed by analysing marker questions from other postgraduate examinations, which should then show an improved performance by UK graduates.

##### iii. Changes in clinical experience

In recent years the working hours of junior doctors have declined, in part due to changes in Government regulations, and clinical experience and hence examination performance may also have declined. In the absence of good measures of clinical experience this hypothesis is difficult to test. There is, however, evidence that the undergraduate clinical experience of UK doctors qualifying in 1996 was lower than that of doctors qualifying a decade earlier [14], and that more recent medical graduates have less knowledge of basic clinical science [15].

##### iv. Changes in undergraduate medical training

Undergraduate medical training in the UK has been continually changing for nearly four decades, dating back primarily to the Royal Commission of 1968 [16], and supported by subsequent recommendations from the General Medical Council [17,18]; new subjects were introduced into the curriculum, and traditional subjects such as anatomy were de-emphasised. Particularly dramatic changes followed the General Medical Council's *Tomorrow's Doctors *[19] of 1993, as a result of which many medical schools introduced major curricular changes, often involving problem-based learning. A number of medical schools also merged, and most medical schools became larger. Although these latter innovations might have caused changes in the knowledge-base of graduates, they are unlikely to explain changes we describe here, which began in 1997 and hence relate to students entering medical school in 1990 or 1991, before the publication of *Tomorrow's Doctors*. A key question concerns whether the standards of undergraduate examinations, both basic medical sciences and finals, have been maintained; however, the patchy use of marker questions, frequent changes in undergraduate examination formats, and the absence of a UK national medical licensing examination make it unlikely that the question can be answered easily. Indeed, the only reliable evidence on the absolute standard of undergraduate training may have to come from performance in postgraduate examinations.

In summary, we have provided evidence of a decline in the performance of candidates taking the MRCP(UK) Part 1 Examination between 1997 and 2001. In addition, as a result of the reliance on norm-referencing, there was also a decline in the standard of those passing the Examination. Criterion referencing is now included as a central part of the MRCP(UK) Examination standard setting process. The reasons for the declining standard of UK graduates are not clear, but on balance are more likely to reflect changes in undergraduate training than changes in postgraduate medical education. More research into the standard of other postgraduate examinations, as well as undergraduate assessments, is urgently needed.

## Competing interests

ICM, JAV, OLD and JM have all been involved with the MRCP(UK) for many years. JM is a paid employee of the MRCP(UK) central office, JAV, OLD, and ICM contribute to the examination academically, and JAV and OLD receive financial compensation for the extensive work that this involves.

## Authors' contributions

The original idea for the study was ICM's, arising from discussions that occurred at Board Meetings chaired by JAV. JM contributed extensive statistical and administrative support to the study, and JAV and OLD contributed to the interpretation of results and the writing of the paper. ICM wrote the first draft of the manuscript and carried out the multivariate statistical analyses. All authors have contributed to the final draft of the paper.

## Pre-publication history

The pre-publication history for this paper can be accessed here:



## Supplementary Material

Additional File 1 Changes in the composition and timing of entries to MRCP(UK) Part 1. Click here for file
