# Supplementary material for: Changes in standard of candidates taking the MRCP(UK) Part 1 examination, 1985 to 2002: Analysis of marker questions
Source: BMC Med. 2005 Jul 18;3:13. doi: 10.1186/1741-7015-3-13 (PMC1185541; doi:10.1186/1741-7015-3-13)
Supplement: Additional File 1 — Changes in the composition and timing of entries to MRCP(UK) Part 1. [file 1741-7015-3-13-S1.pdf]

Supplementary Information

Changes in the composition and timing of entries to MRCP(UK) Part 1.

A concern in Study 1is that the ‘dog-leg’ shown in Figure 2 is potentially an artefact due to some other contemporary change that also occurred in the examination. Although that possibility is made far less likely by the more detailed analyses of Study 2, here we will describe data suggesting that there was not a sudden change in the composition of candidates taking the examination, nor did candidates take the examination at different times in their careers.

i. UK vs Overseas candidates. The black bars in supplementary figure 1 show the numbers of UK candidates taking the examination from 1991 to 2002. The data are only displayed for 1991 onwards since, due to technical problems, not all information, particularly on attempt number, is reliable before that date. All such problems are absent by 1991, which is still sufficiently far ahead of the 1996/7 event of interest to make the data appropriate. There is a very clear cyclical effect, with more candidates taking the examination on diet 1 of a year than on diet 3.This is due to most candidates graduating and gaining provisional registration in May/June of a year, and hence the first diet of MRCP that they can sit is the January diet of the next year but one. There is a small upwards trend in the number of UK candidates taking the examination. However the number of graduates from UK medical schools also increased over the same period, and the proportion of all UK graduates has probably remained fairly stable, meaning that the ‘quality’ is probably unchanged.

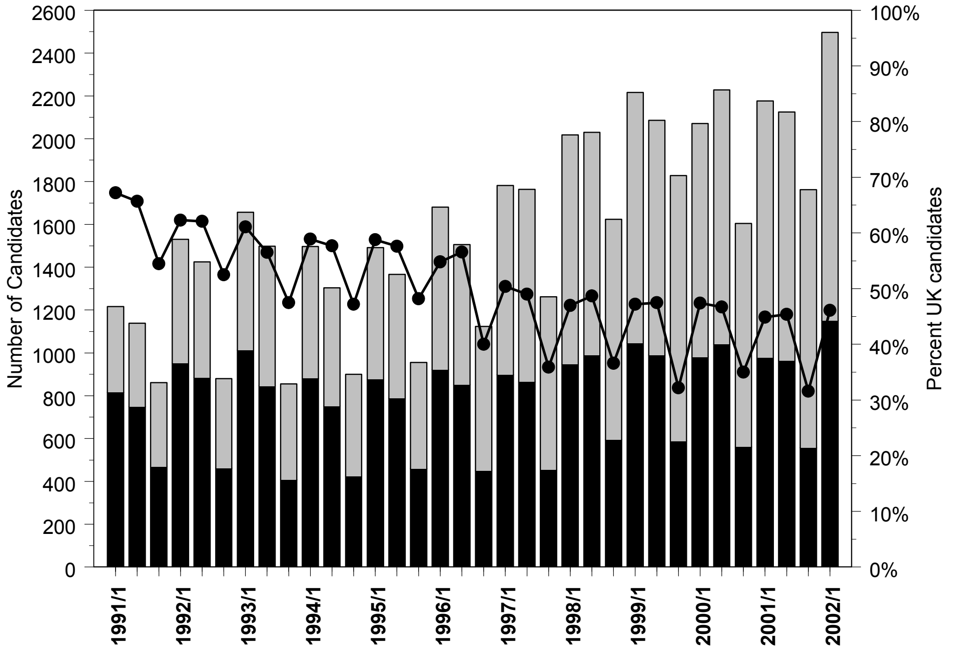

Figure A1: Numbers of UK and non-UK graduates taking MRCP(UK) Part 1

The light grey bars in figure A1 show the numbers of non-UK graduates taking the

examination. These show a much smaller cyclical effect than the UK graduates, and, as will be shown below, this is due to their taking the examination much later after qualification.

The numbers of UK and non-UK graduates do therefore show some changes over the period 1991 to 2002. However none of these show a sudden change at about the date of the dog-leg.

ii. First, second, third, fourth and later attempts at the examination. Supplementary figures A2 and A3 show the proportion of UK and non-UK graduates on their first, second, and later attempts at Part 1. In figure A2 it is clear that in the first diet of the year, most UK graduates are on their first attempt, and the proportion falls rapidly in the second and third diets. Only a small effect is visible in the non-UK graduates, and it is mainly in the early years of the period (see below). The effect of the change in regulations for the 1999/2 diet, allowing fifth and subsequent attempts, is clearly visible, about 5% of UK graduates and 10% of non-UK candidates fitting into that category. The most important result for present purposes is that there is no evidence of any major change in behaviour at around 1996/97.

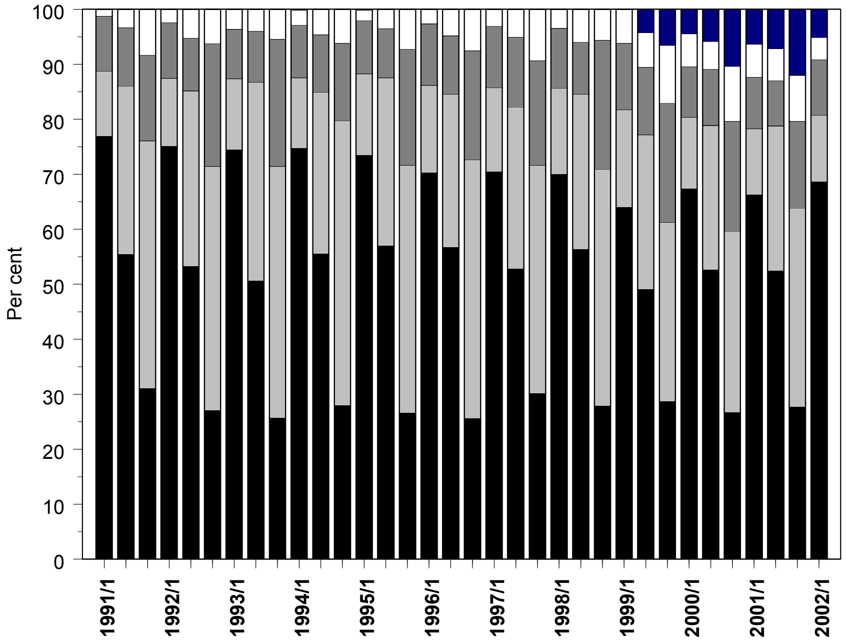

Figure A2: UK graduates – proportion on first, second and subsequent attempts

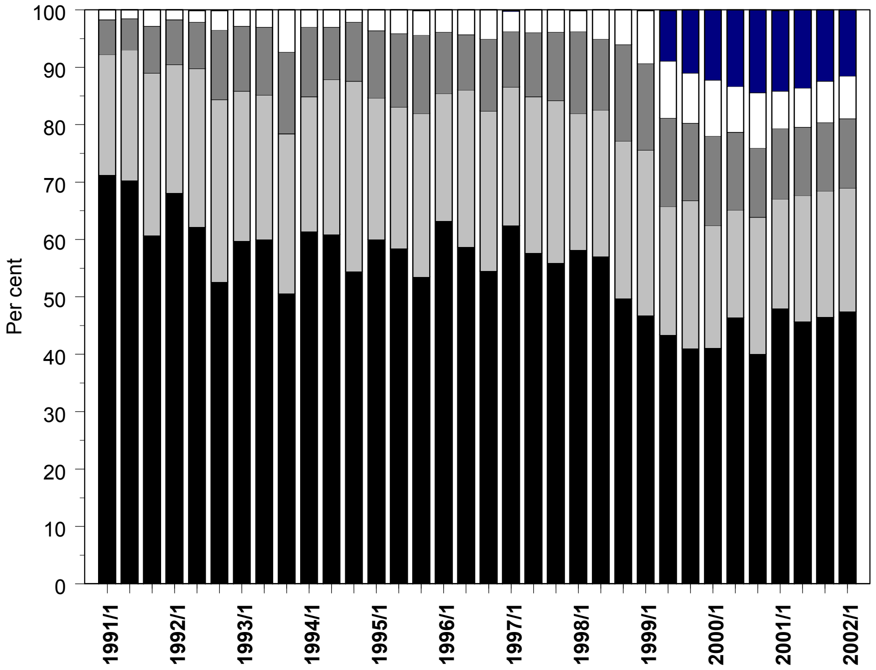

Figure A3: Non-UK graduates – proportion on first, second and subsequent attempts.

iii. Interval between qualifying and taking Part 1. Supplementary figures A4 and A5 show the average time between registration and taking the Part 1 examination, for UK and non-UK graduates at their first, second, and later attempts at the examination. In figure A4 it is clear that most UK candidates are taking the examination for the first time soon after the 18-month deadline, with subsequent attempts following soon after. There is no evidence of any change in this pattern between 1991 and 2002. For non-UK graduates the picture is very different. Figure A5 shows that even in 1991 the first attempt was somewhat later, at nearly two and a half years after graduation, and by 2002 this had risen to about five years after graduation. However subsequent attempts tended to follow quickly on after the first attempt, as in the UK graduates. Again, the crucial feature is that there is no sign of a sudden change in behaviour, around 1996/97.

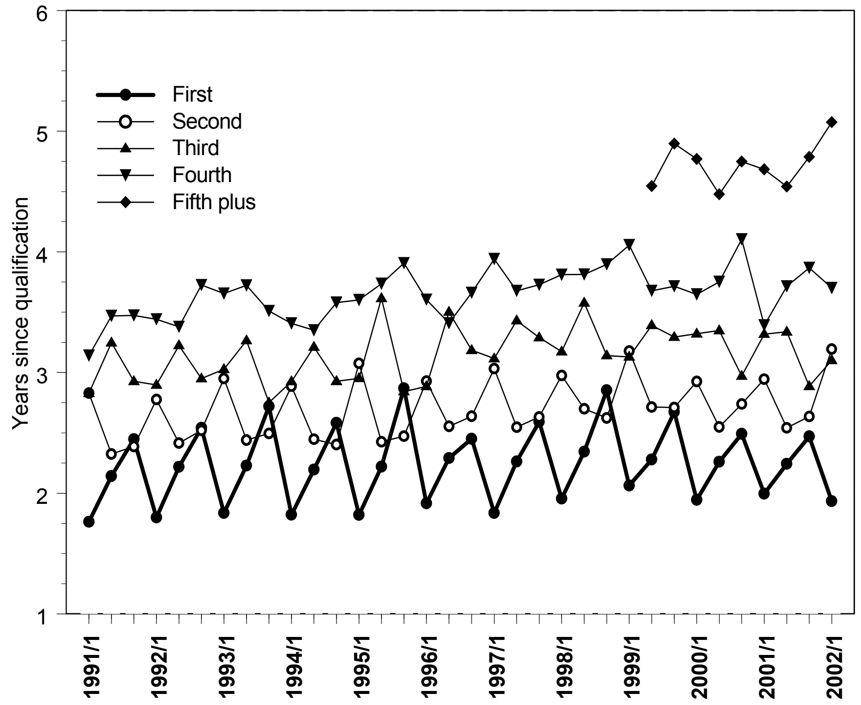

Figure A4: UK graduates – Mean years since qualification at first, second, third, fourth and later attempts.

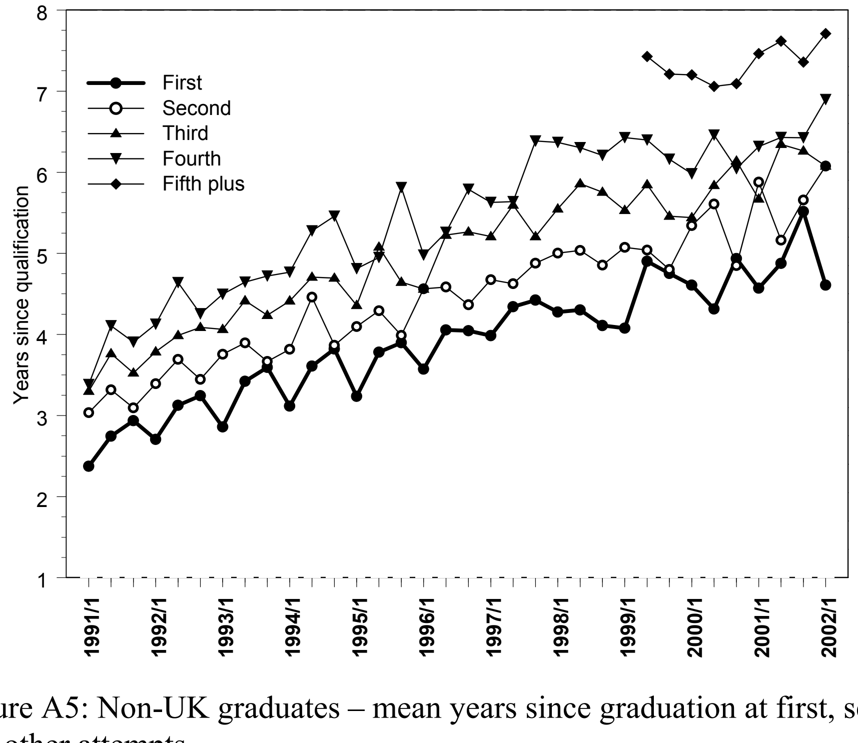

Figure A5: Non-UK graduates – mean years since graduation at first, second and other attempts.
